# Relationship Building between International Healthcare Volunteers and Local Healthcare Providers in Ethiopia: Real-Life Experiences in Low-Income Country

**DOI:** 10.3390/healthcare11131969

**Published:** 2023-07-07

**Authors:** Jiwon Kang, Purum Kang

**Affiliations:** 1 School of Nursing, University of Minnesota, Minneapolis, MN 55455, USA; kang0448@umn.edu; 2 College of Nursing, Woosuk University, Wanju 55338, Jeollabuk-do, Republic of Korea

**Keywords:** volunteers, interpersonal relationships, qualitative research, cultural diversity

## Abstract

Background: The number of international healthcare volunteers in low-income countries that need trained human resources has been increasing. However, migrating to a foreign country requires adapting to its environment and culture. The purpose of this study was to explore the international healthcare volunteers’ experiences in Ethiopia in building relationships with local healthcare providers. Methods: Six participants were enrolled in the study, and data were collected through individual in-depth interviews conducted between September and October 2018. The collected data were analyzed using Colaizzi’s phenomenological method. Results: Ten subthemes emerged from five themes: “facing new situations”, “accepting myself as an outsider in Ethiopia”, “impact on the wall of prejudice”, “adapting to a new culture”, and “positive outlook”. Conclusions: This study shows that international healthcare volunteers in Ethiopia experienced challenges in building relationships with local healthcare providers due to linguistic and cultural gaps. Nevertheless, they strived to accept the culture and play their part as helpers in providing healthcare services.

## 1. Introduction

The World Health Organization has emphasized continuous surveillance and international cooperation to secure health equity [1]. Especially in recent years, cross-country cooperation has been developed further to reduce global inequality in health [2,3]. Substantial financial aid and human resources are continuously deployed to resolve public health problems in low-income countries [4]. Growing interest among individuals and independent organizations has also led to a rise in overseas medical volunteering and the provision of personnel to assist public health-related agencies or hospitals [5]. As international exchanges become more active, the number of individuals serving as international healthcare volunteers in low-income countries has also been increasing. As such, there has been a steady effort to achieve health equity [6]. The Republic of Korea (ROK) also launched the Korea International Cooperation Agency in 1998 [7], which engages in international technological and policy exchanges and dispatches the “ROK overseas emergency rescue team” in response to disasters [8]. In addition, Ethiopia has been designated as one of the least developed countries by the Organization for Economic Cooperation and Development/Development Assistance Committee (OECD/DAC, 2014–2017) and has attracted much attention as a target country for healthcare outreach [4]. In particular, Ethiopia’s rural Amhara region is experiencing many more difficulties than its neighbors in socioeconomic problems and the medical field [9]. Therefore, there are also Koreans present as international healthcare volunteers in Ethiopia.

Migrating to a foreign country for work requires adapting to its unfamiliar environment and cultural diversity. Language, as one primary barrier, reflects the cultural context in which these workers may experience challenges in their basic communication [10]. Communication difficulties are attributable to cultural differences and perceptions about what qualifies as important information [11]. Therefore, in low-income countries, migrant healthcare personnel are required to understand and adapt to new cultural contexts in addition to achieving proficiency in their work. However, since it is not easy to understand a culture that has been shaped over the course of history in a short time, conflicts and different conflict management strategies may emerge when people with different cultural backgrounds interact [12].

In addition, healthcare providers who work at hospitals and human service organizations frequently experience conflicts and emotional stress in building relationships with their colleagues [13]. The inability to cope with stress related to workplace relationships may induce burnout or contribute to turnover intention [14]. Conversely, building relationships is fundamental to existence, and forming amicable relationships can help individuals overcome a myriad of stressful situations [15]. Coworker support with good relationships was negatively associated with job stress in healthcare workers [16]. Clearly, it is important to understand the unique process through which individuals from different cultural backgrounds interact and build work relationships to devise ideal personnel management measures. So, we explore the relationship between Korean international healthcare volunteers with local healthcare providers in Ethiopia. We anticipated that Korean international healthcare volunteers in Ethiopia would also experience increased emotional stability and diminished stress upon building healthy relationships with locals, thereby minimizing conflict.

In fact, in a study on the relationship between coworkers and jobs, it was confirmed that high solidarity among coworkers has a positive effect on job motivation [17]. It has also been shown that peer support in relationships and at work can prevent and manage burnout [18]. However, besides evaluating peer relationships as quantified figures, it is also necessary to comprehensively analyze both the process and the phenomenon. Colaizzi’s phenomenological method [19] makes it possible to understand the phenomenon of an individual’s lived experience by grasping its essential structure. Therefore, it is appropriate to explore the immeasurable process of building relationships and related experiences through self-reflection on one’s lived experiences.

In this context, we aimed to explore and describe the experiences of Korean international healthcare volunteers’ lived experiences in a low-income country and their process of building relationships with local healthcare providers in Ethiopia using Colaizzi’s phenomenological method. We sought an in-depth understanding of the participants’ experiences and theorized that our findings would provide valuable foundational data for developing ideal personnel management strategies to guide migrant healthcare personnel to adapt to their host country and ultimately contribute to providing quality healthcare.

## 2. Materials and Methods

### 2.1. Study Design

This study explores the life experiences of international healthcare volunteers’ in a low-income country and the process of building relationships with local healthcare providers. We used Colaizzi’s method based on Husserl’s philosophical foundation among scientific phenomenological methods to carry out this study [19]. The experiences that international healthcare volunteers formed with local healthcare providers in special situations were described in detail, and the research results were derived from them [20].

### 2.2. Sampling and Ethical Considerations

Participants in this study were international healthcare volunteers from South Korea who had been working for six months or more at a general hospital with 200 beds in Addis Ababa, Ethiopia. They are all licensed healthcare workers from Korea and were willing to voluntarily share their experiences in Ethiopia as international volunteers. They had the ability to express their experiences realistically.

For sampling, we first promoted the purpose and method of the study to the subjects who met the criteria through institutional officials. The subjects who understood the information about the purpose and method of the study and wanted to participate in the study contacted the researcher directly. Additional subjects were recruited using snowball sampling through the participants. To ensure participants’ autonomy, we provided an explanation of the study’s purpose and method and obtained written informed consent. The participants were also informed that they could withdraw from the study at any time, and their anonymity was ensured by using identification numbers assigned to their names during data analysis. This study was approved by the Public Institutional Review Board Designated by the Ministry of Health and Welfare (P01-201810-21-007).

### 2.3. Data Collection

Participants consented to participate after being informed of the purpose and intent of the study (Table 1). To obtain detailed information, participants were selected based on recommendations from university professors and the nursing office of the local hospital in Ethiopia. The data collection period was approximately 25 days, from 20 September to 15 October 2018, and comprised in-depth interviews and participant observation, during which observation notes were used to record participants’ facial expressions, gestures, and attitudes. One of the authors, J.K., conducted the interviews locally in Ethiopia, gathered personal information, had brief conversations, and helped participants become comfortable with the interviewer prior to the interview. Each interview was recorded with the consent of the participant. Interviews were conducted in a quiet place that the participant felt was familiar to them. The main interview questions were “Are there any episodes that were caused by cultural differences?” and “Tell me about your experiences and anecdotes while you were working in the field and encountered local healthcare providers.” Participants were also asked, “What is the most important factor in the relationship with the local healthcare provider in the local area?” An in-depth approach was taken by asking follow-up questions according to the participants’ answers. Each in-depth interview was about one hour and ended when it was judged that the data were saturated because the answers to the questions were being repeated, and no new content appeared.

### 2.4. Data Analysis

Colaizzi’s methodology was used for phenomenological reduction during data collection and analysis [19]. The recorded interview data were transcribed, and the transcripts were read repeatedly with the field notes taken during the interviews to assess the participants’ statements.

P.K., who did not conduct the interviews, transcribed the interview recordings, and J.K., who conducted the interviews, reviewed the transcripts. Subsequently, according to the content analysis method of Graneheim and Lundman [21], meaningful statements about participants’ relationships with patients were identified, similar content was clustered, and redundant statements were deleted. These statements were then rephrased to identify the hidden meanings. The subthemes that emerged from these statements were clustered into abstract themes. The themes were again compared with the raw data for verification. The final analysis results were used to identify the essential structure of the phenomenon of interest, which was relationship building between international healthcare volunteers and local healthcare providers.

### 2.5. Gaining Credible Information

To ensure rigorous qualitative research, we conducted several discussions based on Guba and Lincoln’s criteria [22] during data collection and analysis. To estimate the truth value, we selected participants who were anticipated to be promising candidates for showing the essence of relationships with local health providers and ensured that the interviews were conducted in a relaxed natural environment. After analyzing the results of this study, we asked the participants to see if they matched their experiences. In addition, the validity of the study was also confirmed for international healthcare volunteers who did not participate in the interview. To promote applicability, the results were presented to two volunteers working at a local hospital who did not participate in our study and were asked whether they could relate to the results based on their own experiences. To promote consistency, the entire study process was described in detail, and the results were discussed with one nursing professor with rich experience in qualitative research to achieve a consensus. To promote neutrality, we attempted to bracket our bias during the entire study process and received feedback from a nursing professor and a nursing PhD to ensure that our subjective thoughts were not involved in the interpretation process.

## 3. Results

The essence of the international healthcare volunteers’ experience is described in 5 themes with 10 subthemes (Table 2).

### 3.1. First Theme: Facing New Situations

This theme was concerned with participants’ preparedness for the new environment and the situations they would have to face when meeting local healthcare providers for the first time. Since beginning to serve as international healthcare volunteers in a new country, they encountered cultural differences and contemplated ways to build relationships with and present themselves to local healthcare providers. This theme identified the subthemes of preparedness prior to the first interaction with people from other cultural backgrounds and the positive emotions underlying the preparation for the meeting. These subthemes included “Readying myself for interpersonal engagement” and “Identifying common emotions.”

### 3.2. Readying Myself for Interpersonal Engagement

Participants expressed their belief that it is necessary to be mindful and prepared to reduce prejudice in a new environment and to maintain language skills, friendly expressions, and behaviors as international healthcare volunteers. In particular, they expressed that language serves as a catalyst for getting closer to each other and also makes the atmosphere naturally friendly. They also mentioned that with the right attitude and mindset, they were able to remain relaxed when communicating with the local healthcare providers, frequently meet with them, and naturally grow closer over time.

“I think we become comfortable with each other when we think that we are similar and it’s okay to be together. Then they will have no problem accessing me.” (The participant showed a memo with Amharic words learned from a local healthcare provider with an expression full of interest).

(Participant 1)

### 3.3. Identifying Common Emotions

While in Ethiopia, participants encountered diverse local healthcare providers and experienced an array of responses. They said that, as these experiences accumulated, they were able to more easily understand the locals’ behaviors. Thus, they paid attention and acted according to the insights gained from local healthcare providers’ responses. In particular, they tried to express positive feelings and gratitude using the local language to win their hearts.

“I think nurses are generally quick-witted. If it seems I don’t know something, they’re willing to teach me how to deal with a problem. At that time, I was not used to the local language, but I think I tried to use the local language a lot when speaking, to show them a positive attitude and gratitude. I asked questions in their language as much as possible and we taught each other.”

(Participant 3)

### 3.4. Second Theme: Accepting Myself as an Outsider in Ethiopia

This theme described the participants’ experiences as they realized that they were outsiders in Ethiopia and discovered their identity in the new country during their struggles with local healthcare providers. They stated that those experiences helped them think about their position, accept it, and adopt an open attitude to identify others’ needs and offer adequate help. This theme included the following subthemes: “Recognizing my location,” and “Putting myself in their shoes”.

### 3.5. Recognizing My Location

The participants described the importance of clearly understanding their positions as international healthcare volunteers when communicating and interacting with local healthcare providers. The participants mentioned that if they focus on the fact that they have come from a country with a better environment to help improve healthcare in a low-income country, they remain narrow-minded, which clouds the essence of the volunteering work they pursue.

“We’re international healthcare volunteers. We think we are in a superior position when we provide healthcare services. When we think of our country, we believe it’s superior to Ethiopia. It is a tremendous power trip to look down on them.”

(Participant 4)

### 3.6. Putting Myself in Their Shoes

The participants stated that instead of thinking that they had to impart new technology and knowledge to the local hospital as international healthcare volunteers, they needed to develop an open attitude to identify and help with what the local healthcare providers really wanted and needed. The participants mentioned that local healthcare providers needed a chance to accept the massive change that occurs when they accept outside personnel aid, and that they may be resistant to this change. Participants further stated that local healthcare providers are unaware of the need for outside aid, even though they know that the technology used by international healthcare volunteers is much more developed, and that they need time to accommodate it, even when they accept the need for it.

“I’ve come here and taught a few things, and these things may cause misunderstandings and may be completely unnecessary for them. First, you need to grow closer to them before teaching and helping out. I think just giving advice with complete disregard for their feelings of not wanting it, puts them on their guards and is viewed negatively by them. It’s important to be ready to gladly help when they are in need, and not undermine them and think that they need change”

(Participant 2)

### 3.7. Third Theme: Impact on the Wall of Prejudice

This theme includes experiences of conflict the participants encountered in new situations at the local hospital and communicating with local healthcare providers. Since they met local healthcare providers frequently, they experienced phases of uncertainty while developing relationships with them. Moreover, although they expressed difficulties in communication, they tried to adapt. This theme included the following subthemes: “Facing prejudices by locals and being challenged” and “Undergoing trial and error”.

### 3.8. Facing Prejudices by Locals and Being Challenged

The participants mentioned that while trying to grow closer to the local healthcare providers, they experienced a defensive and unwelcoming attitude directed at them. In the process of forming a relationship with local healthcare providers, participants’ unilateral advice only raised the guard of locals.

“They were defensive toward me. When they made a mistake, they were busy covering it up, and they would signal to each other to quickly, quickly cover it up because I’m here.”

(Participant 5)

### 3.9. Undergoing Trial and Error

Participants stated that they realized that the locals were not necessarily expecting them to be an indispensable resource, contrary to their belief. After reevaluating their feeling that they must meet the locals’ expectations, the participants felt it was necessary to be sincere in liaising with the locals over time and to exercise care while offering help. Participants realized that they should not help in the same way as in Korea because of cultural differences. It took a long time to deliver the new content.

“At the hospital, when I say something like ‘we do it like this in Korea’ to help them, they were kind of blocking me off, and it caused misunderstandings and made them more resistant toward accepting us… I came to think that if we want to teach something, it should be done slowly and over a long period of time. Even if there was something that needed to be said, it was important to choose the right time to talk about it.”

(Participant 2)

### 3.10. Fourth Theme: Adapting to a New Culture

The participants realized that adapting to the new culture and adhering to its rules is important when building relationships. They emphasized that accepting the local environment and culture and being non-judgmental toward the locals or the country is important. This theme included the following subthemes: “Let go of all prejudices and bias and emotionally get along with the locals” and “Embracing and recognizing the circumstances/culture while comprehending the intricacies of the local ethos”.

### 3.11. Let Go of All Prejudices and Bias and Emotionally Get along with the Locals

The participants reported that by spending time with their local healthcare provider, they learned to be considerate of each other, felt closer psychologically, and found strengths in their relationship. They described that this phenomenon developed as they overcame their prejudices and engaged in increased interaction with each other. The participants also indicated that frequent communication helped them improve understanding and relax in each other’s presence.

“Over time, we learned to be considerate and caring of one another. We tried to remain punctual since that is a value and norm in our country; however, it may not be a problem at all in this country. Through those experiences, I again became aware of my stance about cultural aspects; it really helped me understand the people better.”

(Participant 3)

### 3.12. Embracing and Recognizing the Circumstances/Culture While Comprehending the Intricacies of the Local Ethos

The participants mentioned that accepting the local situation and culture is the most important aspect of building favorable relationships and strengthening their bonds with local healthcare providers. They mentioned that recognizing cultural differences and behaving accordingly played a crucial role in building relationships and helping fulfill locals’ needs.

“In Korea, I lived within an organization. I’m familiar with that organization and my roles are clear, and I have responsibilities. So, I have to do my share no matter what; but here, if you think about it, in a sense… although it’s kind of vague, but I think I’ve experienced that I’m not here to actually do something, but I am here because I am needed.”

(Participant 2)

### 3.13. Fifth Theme: Positive Outlook

The participants mentioned that the process of building relationships with the local healthcare providers by interacting with them despite occasional conflicts, and gradually forming an opinion of them, was important. They mentioned that it was important to foster a relationship in which they could understand and be considerate of one another over time. This theme included the following subthemes: “Discovering and recognizing strengths” and “The significance of fostering a rapport.”

### 3.14. Discovering and Recognizing Strengths

As the participants overcame their prejudices and engaged in frequent interaction with local healthcare providers, they recognized the importance of accepting a situation as important, rather than responding to the situation based on preconceived notions. In addition, they mentioned that the most important thing was not being helpful to the locals and forcing help onto them, but rather spending time with the locals and understanding them.

“I realized that instead of thinking that I should do something, I just needed to spend time with them every day. Even if I was not good at something, there were tasks that these people were good at. The criterion for being the least developed country is only a standard set against all countries, and it was not set by them. These people are immensely happy about little things, instead of focusing on their living conditions. They are happy over a piece of candy.”

(Participant 3)

### 3.15. The Significance of Fostering a Rapport

The participants stated that the most important factor in fostering relationships with local healthcare providers was an accepting and acknowledging attitude. They stressed the importance of fostering relationships in which they understood one another by spending more time together and considering the other person’s needs from their perspective. When they start to understand them, the relationship develops toward friendship, and they say they experience a change in their attitude toward them.

“So, instead of trying to teach something, just first be a friend… If we are demanding right from the first interaction and order them to do something, they might resist and wonder, ‘Who are they to treat me like this?’ So, we shouldn’t approach them like that at first. When I began to think that I came here to understand them, to help them if they needed something, then I think these people began to open up to me and changed their attitudes toward me.”

(Participant 6)

## 4. Discussion

In this study, we used the phenomenological method to gain an in-depth understanding of the experiences of international healthcare volunteers in Ethiopia in building relationships with local healthcare providers. A total of 5 themes and 10 subthemes were identified.

In this study, participants experienced acculturation as they gained exposure to the culture and built relationships with the locals in a new situation, and accepted themselves as outsiders. They mentioned that they fostered positive relationships with local healthcare providers as they strived for acculturation in their host country. Acculturation is the process by which an individual is exposed to a new culture and successfully adapts to it [23]. Learning the language and expected behaviors of the host culture is the key to acculturation; therefore, these parameters are sometimes used to measure the level of acculturation [11], and failure to achieve these goals increases acculturation stress, thus hindering adaptation. Previous research exploring local healthcare providers’ perceptions and attitudes toward international healthcare volunteers showed that international healthcare volunteers had different expectations compared with their daily working environment [24]. Thus, it would be effective to provide international healthcare volunteers with education about language and cultural behaviors regarding the host country to reduce acculturation stress and promote faster adaptation.

Experiencing prejudice and discrimination from locals and encountering confusing rules is part of the process of adapting to a new culture [25]. International healthcare volunteers in our study accepted and adapted to the confusing rules and local culture through trial and error. A study on the cultural adaptation of international students in Turkey reported that it was important for the students to continuously communicate with local institutional managers to adapt systematically [26]. This report is consistent with our finding that continuous communication and trial and error help reduce prejudice or misunderstandings. It is not enough to simply provide international healthcare volunteers with educational programs for cultural adaptation; providing continuous communication channels also helps cultural adaptation. In order to operate a sustainable outreach program, it would be helpful to evaluate and manage whether international healthcare volunteers understand or accept local culture.

When confronted with a new environment, international healthcare volunteers think in terms of their experiences and circumstances in their home country. Therefore, it also shows the appearance of looking at the situation with prejudice and trying to solve it. However, in such a situation, if they accept the lifestyle and rules of local healthcare providers, international healthcare volunteers may feel that it can be easier to adapt to the local situation. Previous studies showed that international healthcare volunteers first need to understand and accept local culture to play health-related roles [27]. Thus, accepting local cultural norms is essential to continuing outreach programs in low-income countries [28]. Therefore, “Impacting the wall of prejudice” and “letting go of all prejudices and biases and emotionally getting along with the locals” may seem a bit conflicting, but in terms of relationship building, both can appear.

Trust relationships with colleagues could be a job motivator and affect the quality of work in healthcare units [17], which also applies to healthcare volunteering in this study. In cross-cultural environments, constructing trust relationships with local colleagues requires time and patience, which the participants in this study understood. Therefore, developing empathy while spending time with locals helps maintain a continuous outreach program without volunteers dropping out.

In our study, international healthcare volunteers in a low-income country strived to accept the acculturation process and their role as helpers while attempting to build rapport with local healthcare providers. With growing emphasis on the importance of health equity, there is significant interest in providing healthcare support in low-income countries. Not only has the international movement of healthcare clinicians increased, but the number of volunteers dispatched to hospitals in other countries has also increased [2,3]. Therefore, the number of international healthcare volunteers in low-income countries is expected to increase further. International healthcare volunteers have to deal with issues related to the cultural adjustment of work in a new country and may have difficulty establishing relationships with local healthcare providers. This affects the effectiveness of volunteer work, and eventually, depending on whether or not they have adapted, their return to their home countries may be brought about earlier [24]. In addition, compared to high-income countries, low- and middle-income countries such as Ethiopia have poor health states and clinical outcomes due to low accessibility to healthcare facilities, including healthcare providers [29]. Therefore, if international healthcare volunteers are integrated into the local context over a long period of time and have a culture-oriented approach, accessibility to health services will also improve and lead to the enhancement of clinical outcomes. Thus, international healthcare volunteers should prepare a mindset to break away from the attitudes they practice in their home countries and work toward understanding and accepting the ways of the local medical staff.

We acknowledge the limitations of this study, because we included only a small number of subjects. Participants in this study had to be preceded by the special conditions of long-term international healthcare volunteering experience; thus, the population size was not large, and recruitment was limited. Despite this limitation, we included healthcare volunteer subjects from various occupations, ages, and careers. In addition, we should note the originality of this study in including participants who have performed international healthcare volunteering for a long time. In addition, based on in-depth interviews, we identified the processes international healthcare volunteers underwent to establish relationships in new cultural contexts. Based on this study, more research using various methods will be required to understand the process of international healthcare volunteers adapting to local organizations and building relationships with coworkers.

## 5. Conclusions

The results of this study show that international healthcare volunteers in Ethiopia experienced challenges in building relationships with local healthcare providers due to linguistic and cultural gaps, but they strived to accept the local culture and play their part as helpers in providing healthcare services.

International healthcare volunteers who reached out to low-income countries for medical assistance initially faced new situations in establishing relationships with local healthcare providers and recognized themselves as strangers in a different culture. Afterward, they encountered prejudice as a foreigner, went through trial and error, and formed a closer relationship with the local healthcare providers through the process of accepting a new culture. They tried to work as helpers in cooperation with local healthcare providers, not just providers that deliver medical skills. During this process, we discovered that it is important to constantly find the positive side of a situation, even if there are difficulties due to conflicts due to cultural differences.

With a growing emphasis on health equity and globalization, the number of international healthcare volunteers is expected to rise. We suggest using our findings as baseline data to develop effective acculturation strategies for international healthcare volunteers. Since this study was conducted on healthcare providers at a single hospital, subsequent studies should consider several other countries and types of hospitals to ensure transferable results. In addition, although this study explored the process of establishing peer relationships between international healthcare volunteers and local healthcare providers, future research on the relationship and trust between international healthcare volunteers and patients with enhanced cultural competence would be meaningful.

## Figures and Tables

**Table 1:** Participants’ characteristics.

Age (Years)	Gender	Marriage Status	Education	Current Position	Working Department	Career in Korea	Career in Ethiopia
30	Woman	Single	University graduate	Nursing staff	Internal medicine Nursing office	6 years	6 months
34	Woman	Single	University graduate	Nursing staff	Nursing office	11 years 5 months	1 year 3 months
44	Woman	Married	University graduate	Clinical pathologist	Department of diagnostic Tests	17 years	1 year
58	Man	Married	University graduate	Doctor	Neuro-surgery	25 years	1 year 7 months
64	Man	Married	Graduate school graduate	Doctor	Neuro-surgery	35 years	1 year 2 months
73	Woman	Married	University graduate	Nurse (Director of nursing department)	Nursing office	45 years 9 months	4 years 10 months

**Table 2:** Themes and theme cluster of health volunteers’ experience of relationship building in a low-income country.

Theme Clusters	Themes
Readying myself for interpersonal engagement	Facing new situations
Identifying common emotions	
Recognizing my location	Accepting myself as an outsider in Ethiopia
Putting myself in their shoes	
Facing prejudices by locals and being challenged	Impact on the wall of prejudice
Undergoing trial and error	
Letting go of all prejudices and biases and emotionally getting along with the locals	Adapting to a new culture
Embracing and recognizing the circumstances/culture while comprehending the intricacies of the local ethos	
Discovering and recognizing strengths	Positive outlook
The significance of fostering a rapport	

## Data Availability

Not applicable.
